# Exploration of SOCS3 and HSPA1A in PANoptosis during acute myocardial infarction and chronic kidney disease

**DOI:** 10.3389/fcimb.2026.1844856

**Published:** 2026-07-22

**Authors:** Guoyong Liu, Hongli Xian, Yehao Xu, Jun Ma, Jingyi Huang, Lili Ye

**Affiliations:** 1 Department of Cardiovascular Medicine, GuangDong Engineering Technology Research Center of Biological Targeting Diagnosis, Therapy and Rehabilitation, The Fifth Affiliated Hospital, Guangzhou Medical University, Guangzhou, Guangdong, China; 2 Department of General Practice, GuangDong Engineering Technology Research Center of Biological Targeting Diagnosis, Therapy and Rehabilitation, The Fifth Affiliated Hospital, Guangzhou Medical University, Guangzhou, Guangdong, China

**Keywords:** acute myocardial infarction, chronic kidney disease, immune microenvironment, PANoptosis, single-cell RNA sequencing

## Abstract

**Introduction:**

Chronic kidney disease (CKD) is an independent risk factor for acute myocardial infarction (AMI), but the mechanisms underlying their comorbidity remain unclear. As a newly described inflammatory form of programmed cell death, PANoptosis may play a key role. This study aimed to identify shared pathogenic mechanisms and key targets linking the two diseases.

**Methods:**

Mendelian randomization analysis was performed to assess the causal relationship between CKD and coronary artery disease (CAD). Shared genes among AMI, CKD, and PANoptosis were identified, followed by functional enrichment analysis. Diagnostic performance of key genes was evaluated using independent cohorts. Single cell sequencing was applied to localize gene expression at the cellular level. In vivo animal experiments were conducted to validate the functional role of the candidate gene SOCS3 in post MI PANoptosis and cardiac function.

**Results:**

Mendelian randomization confirmed that CKD is a significant risk factor for CAD. We identified 12 genes shared by AMI, CKD, and PANoptosis, and determined SOCS3 and HSPA1A as key diagnostic genes. Both genes displayed strong diagnostic performance (e.g., AUCs > 0.85 for AMI) in independent cohorts. Functional analysis showed enrichment in pathways including JAK STAT signaling and apoptosis. Single-cell sequencing localized high expression of these genes to monocytes and endothelial cells. Animal experiments demonstrated that knockdown of SOCS3 significantly inhibited expression of key PANoptosis molecules such as CASP1, CXCL10, and ZBP1 after MI, reduced inflammation, and improved cardiac function.

**Discussion:**

PANoptosis was the central pathological link connecting CKD and AMI. As shared biomarkers and intervention targets, SOCS3 and HSPA1A provided new directions for precise diagnosis and treatment of cardiorenal comorbidities.

## 1 Introduction

Coronary artery disease (CAD) has become the leading cause of global mortality, causing 15 million deaths in 2016 alone (Roth et al., 2018). Although CAD death rates have decreased recently, public health impacts and economic burden remain substantial (Duggan et al., 2022). CAD is mainly caused by atherosclerosis from lipid deposition and inflammation, leading to plaques that can cause angina, heart attacks, or acute myocardial infarction (AMI) (Bhatnagar et al., 2015; Bhatt et al., 2022). Clinically, AMI is defined by high cardiac biomarkers that indicate myocardial injury and evidence of ischemia (Thygesen et al., 2018). AMI is typically caused by rupture or erosion of vulnerable plaques, leading to acute thrombus formation and coronary artery occlusion, which result in myocardial ischemia, cellular injury, and ultimately myocyte death (Libby, 2013). Current treatments such as statins and stent implantation often fail for some patients. Therefore, new preventive and adjunctive targets should be pursued.

Chronic kidney disease (CKD) is defined primarily by a persistent decline in glomerular filtration rate (GFR) (Lameire et al., 2021; Romagnani et al., 2025). CKD and CAD, particularly AMI, are closely and complexly linked (Dilsizian et al., 2021; Shin et al., 2025). This relationship shows shared risk factors like hypertension and diabetes, along with common mechanisms of chronic inflammation and oxidative stress (Wanner et al., 2016; Lai et al., 2021). Reduced renal function in CKD leads to uremic toxin accumulation, activating the immune system and causing low-grade inflammation, which raises inflammatory cytokines and reactive oxygen species, promoting vascular dysfunction and atherosclerosis (López-Novoa et al., 2011; Arreola Guerra and Martinez-Martinez, 2025). In inflammatory environments, plaque stability weakens, increasing vulnerability and leading to thrombus formation and AMI (Roth et al., 2018). CKD disturbances in calcium-phosphorus metabolism and vascular calcification raise cardiovascular event risk (Bhatnagar et al., 2015; Duggan et al., 2022). Therefore, CKD amplifies CAD risk beyond traditional factors due to systemic vascular injury and thrombotic tendency from inflammation and oxidative stress (Sarnak et al., 2003). Notably, CKD is an important and independent risk factor for CAD and AMI, but the specific underlying mechanisms have not been fully elucidated.

In recent years, multi-omics integration strategies have emerged as essential tools for deciphering the molecular mechanisms of complex diseases, revealing links between organ systems and identifying novel targets (Emdin et al., 2018; Stuart et al., 2019; Suhre et al., 2021). Building on their successful application to biomarker studies in cardiorenal syndrome and CAD (Nikpay et al., 2020), this study used a four-stage integration framework Mendelian randomization (MR), transcriptome-wide association analysis, single-cell heterogeneity analysis, and experimental validation—to systematically investigate the shared molecular mechanisms between CKD and AMI.

PANoptosis is a distinct, physiologically significant form of inflammatory programmed cell death (PCD) regulated by the PANoptosome complex and exhibiting features of pyroptosis, apoptosis, and necrosis (Christgen et al., 2022). After inflammasome assembly and activation, gasdermin (GSDM)–dependent pores form in the plasma membrane, a hallmark of PANoptosis (Liu et al., 2021). As an emerging mode of cell death, PANoptosis plays an important role in the pathogenesis of AMI and CKD. The primary objective of this study was to identify key genes associated with AMI, CKD, and PANoptosis to reveal shared pathogenic mechanisms and potential therapeutic targets. By integrating genetic, transcriptomic, and machine learning analyses, this study aimed to clarify the complex relationship between these two common diseases and to provide new insights for preventing and treating AMI in patients with CKD.

## 2 Methods

### 2.1 Data source

To evaluate the effect of CKD on CAD, we performed an MR analysis using CKD as the exposure and CAD as the outcome. The Genome-Wide Association Study (GWAS) summary data for CKD were obtained from ebi-a-GCST003374 (population: European), and GWAS data for CAD were obtained from ieu-a-7 and ieu-a-9 (population: Mixed).

Transcriptome data were downloaded from the GEO database (http://www.ncbi.nlm.nih.gov/geo/), namely, AMI datasets GSE59867 and GSE62646 (Zhang et al., 2023) and CKD datasets GSE66494 (Pei et al., 2025). GSE59867 contained 111 ST-segment elevation myocardial infarction (STEMI) patients and 46 CAD patients; GSE62646 contained 28 STEMI patients and 14 CAD patients; GSE66494 contained 53 CKD patients and 8 normal control subjects. GSE123342 (Tang et al., 2026) dataset (65 AMI patients and 22 CAD patients) was used to evaluate hub genes.

Single-cell data for AMI were obtained from GEO under accession numbers GSE184073 (Yoshida et al., 2026), GSE121893 (Zhou et al., 2025), and GSE180678 (Song et al., 2022). GSE184073 includes data for one AMI patient and one CAD patient; GSE121893 has data for two CAD patients, and GSE180678 has data for one AMI patient. Single-cell data for CKD was obtained from GEO under accession number GSE199711 and contains data for three CKD patients and two healthy individuals. Dataset characteristics are summarized in **Supplementary Table 1**.

The GEO datasets were chosen according to established criteria for transcriptomic meta-analyses. The relevance of tissue was ensured by selecting peripheral blood mononuclear cells (PBMC) or whole blood, which reflect systemic inflammation in CKD-AMI pathogenesis. A minimum of 20 samples per group was required to maintain statistical power. Platform consistency was achieved by using Affymetrix U133 Plus 2.0 arrays, facilitating cross-study integration. All datasets were independently validated in peer-reviewed studies.

PANoptosis-related genes were searched from GeneCards (http://www.genecards.org/) using the search term “PANoptosis.” Using a relevance score cutoff of >3 yielded 2,535 PANoptosis-related genes.

### 2.2 MR analysis

We used MR to determine causality between CKD and CAD. IEU OpenGWAS summary statistics were used to select the single nucleotide polymorphisms (SNPs) associated with CKD and CAD.

We used five complementary methods of MR to determine the causality between CKD and CAD. We used inverse variance weighting (IVW) (Lee, 2019), MR Egger (Zheng et al., 2021), weighted median (Bowden et al., 2016), simple mode (Li et al., 2022) and weighted mode (Hartwig et al., 2017). The IVW method was used as the primary statistical analysis, and significance was *p* < 0.05. The odds ratio (OR) was used to quantify associations between risk and protective factors. Weighted median and MR-Egger methods were used to evaluate the causality of genetic susceptibility to CKD for the CAD risk.

Heterogeneity between instrumental variables (IVs) was evaluated using the mr_heterogeneity function in TwoSampleMR and the mr_pleiotropy_test function to detect potential bias from pleiotropy. The leave-one-out function performed leave-one-out sensitivity analysis of the effect of individual genetic variants on the overall MR estimate. Finally, MR-Steiger filtering was used to determine the direction of the exposure and outcome.

### 2.3 Identification of differentially expressed genes

After processing the data for each disease, we compared the AMI and the CKD data using the Linear Models for Microarray Data (LIMMA) package in R version 4.3.2. We computed differentially expressed genes (DEGs) between disease and control groups. The DEG threshold for AMI was adj.P.Val < 0.05 and adj.P.Val < 1 for CKD. We visualized the differential analysis results for each group using heatmaps and volcano plots, in blue (low expression) and red (high expression) in both plot types.

### 2.4 Weighted gene co-expression network analysis (WGCNA)

WGCNA is a systems biology method to build gene coexpression networks and identify modules associated with AMI and CKD. The R package WGCNA clusters genes into modules according to co-expression similarity across samples and links modules to external clinical traits. By mapping functional networks to clinical traits, WGCNA can reveal biomarkers and identify new molecular drivers. Normalized mRNA expression data (calculated using the R package LIMMA) were used as input for WGCNA to detect gene coexpression modules and assess correlations between modules and clinical status (AMI/CKD vs. control).

We identified sample outliers by hierarchical clustering using the R package gplots, evaluated soft-thresholding power values 1–20 with pickSoftThreshold, converted the correlation matrix with the chosen β value into an adjacency matrix and topological overlap matrix, constructed a hierarchical clustering tree with average linkage on TOM, identified gene modules using the dynamic tree cut algorithm (minModuleSize = 50), and assessed correlations between gene modules and clinical traits (AMI or CKD) using the Pearson correlation coefficient.

Modules with the highest correlations [MEcyan (0.62, GSE59867), MEturquoise (0.65, GSE62646), and MEpink (0.89, GSE66494)] for CKD. As several modules had strong associations with AMI and CKD, genes were also categorized by gene significance (GS) and module membership (MM). Genes most closely related to the diseases were selected by hub modules with |MM| > 0.8 and |GS| > 0.5 for AMI and CKD groups.

### 2.5 Identification of genes shared with PANoptosis and functional enrichment

We combined module genes identified by WGCNA with DEGs to identify common key genes involved in AMI and CKD pathogenesis and intersected these genes with PANoptosis phenotypes to obtain DEGs involved in AMI, CKD, and PANoptosis pathogenesis. In order to measure the biological functions and signaling pathways of the core genes, we used the clusterProfiler package to measure Gene Ontology (GO) and Kyoto Encyclopedia of Genes and Genomes (KEGG) enrichment studies. The bar chart showed significant functional enrichment (*P* < 0.05).

### 2.6 Feature selection using two well-known machine learning algorithms and prediction performance assessment in validation cohorts

We used machine learning algorithms LASSO (Least Absolute Shrinkage and Selection Operator) and random forest (RF) to screen candidate diagnostic genes of AMI, CKD, and PANoptosis. First, we input 12 genes previously known as shared by AMI and CKD separately into LASSO for each disease group. We used the R package glmnet to construct a 10-fold cross-validation regression model, set the family parameter to a binomial parameter, and selected the best lambda value via lambda.1min. Logarithmic (λ) trajectory of 12 LASSO coefficients was plotted and the partial likelihood deviance curve was generated. Second, the optimal 1se value (1-SE) was calculated for the minimum standard. Finally, R package randomForest was used to rank and classifying important genes with the RF algorithm.

We applied decision tree and RF analysis to identify the most important variables and filter shared genes from disease genes. First, we constructed a RF model with 500 trees in the discovery cohort and used cross-validation error to determine the optimal tree number. We ranked genes by importance and plotted the top 10 for each disease group using a significance threshold of 0.9. Both algorithms intersected the results. The Venn diagram shows four genes in the AMI group and four genes in the CKD group, and a combination of common genes yielded two target genes for disease diagnosis. We used the packages pROC and ggplot2 for visualizing the diagnostic performance of the two genes in the discovery cohort and selected GSE123342 (AMI) for validation. We downloaded raw data from the GEO database and normalized it with the RMA package. We visualized the expression of genes using box plots and calculated the AUC area in the validation cohort.

### 2.7 GSEA implementation for single diagnostic gene

After finding the diagnostic genes, we used the clusterProfiler package to perform single-gene of Gene Set Enrichment Analysis (GSEA) for each diagnostic gene in both groups. GSEA compares biological signaling pathways between disease and control groups. Gene sets were downloaded from MSigDB (c5.all.v2023.2.Hs.symbols.gmt). The enrichment map lists top nine activated and inhibited pathways for each gene in each disease group.

### 2.8 Immune cell abundance

We ran CIBERSORT analysis on each disease sample to compute relative immune cell proportions. According to the CIBERSORT website (http://cibersort.stanford.edu/) LM22 has 22 signatures, and using 1,000 iterations of CIBERSORT, we quantified 22 immune cell types according to LM22 signatures. For downstream analyses we kept samples with CIBERSORT *P*-values of 0.05. We normalized the CIBERSORT output estimates to 1 for each retained sample so that comparisons can be made across immune cell types and datasets. We visualize results using the R packages corrplot, vioplot, and ggplot2. Spearman nonparametric correlation analysis was used to compare immune-infiltrating cells to diagnostic target biomarkers.

### 2.9 Single-cell data preprocessing and gene expression analysis

We first downloaded raw single-cell data and processed it using the Seurat package (4.4.1). We normalized the expression matrix by computing per-cell UMI proportions and number of mitochondrial gene counts and then used Seurat’s FindVariableFeatures to select the top 2,000 highly variable genes (HVGs) to integrate, using batch effects (ifnb). These HVGs were used for principal component analysis (PCA), and cell populations were identified as clustering 1.0 and projected into two dimensions in RunTSNE.

All cells in ACS were clustered into 12 groups based on expression data, whereas cells in CKD were clustered into 18 groups. We used established marker genes and assigned cells to different populations. We used Seurat’s DotPlot function and feature plot function together to show SOCS3 and HSPA1A expression patterns in these populations. We also performed differential expression analysis using Seurat’s FindMarkers function using average fold change (log_2_FC) ≥1 and *p* < 0.05. These methods ensured accurate identification of cell types and DEGs.

### 2.10 Ethical statement on animal experiments and construction and injection of AAV9 vectors

Animal experiments were conducted according to the Guide for Laboratory Animal Care and Use (NIH Publication 85-23, revised in 1996) and approved by the Animal Ethics Committee of Guangzhou Medical University (P2025-25049). Male C57BL/6J mice, 8–10 weeks old and 22–25 g, were obtained from Vitalstar Biotechnology Co., Ltd. (Beijing, China). Mice were housed in a specific pathogen-free (SPF) facility on a 12h light–dark cycle and were ad libitum fed with food and water.

### 2.11 Mouse myocardial infarction model

Mice were anesthetized with 2% isoflurane using a respiratory anesthesia machine, R500 Reagened Life Science, Shenzhen, China. Left thoracotomy was performed in the fourth intercostal space to open the heart. The left anterior ascending coronary artery was ligated with 7–0 silk sutures (Ningbo Medical Needle Co. Ltd., China). Myocardial ischemia was confirmed by rapid blanching of the anterior wall. Mice in the sham group were treated with the same procedure without ligation.

### 2.12 Echocardiography

Four weeks after myocardial infarction, cardiac function was evaluated with a high-resolution imaging system (Vevo 2100, FUJIFILM VisualSonics, Toronto, Canada) with MS400 transducer. Mice were lightly anesthetized with 1.5% isoflurane. M-mode images were taken at the papillary muscle level. Left ventricular ejection fraction (LVEF) and fractional shortening (LVFS) were calculated using VevoLAB software.

### 2.13 RNA extraction and quantitative real-time PCR (qPCR)

Total RNA was extracted from left ventricular tissue using TRIzol reagent (Catalog No. 15596026, Thermo Fisher Scientific, Waltham, USA). RNA concentration and purity were measured with NanoDrop 2000 spectrophotometer (Thermo Fisher Scientific). Reverse transcription was performed with the PrimeScript™ RT kit (with gDNA Eraser, Catalog No. RR047A, Takara, Shiga, Japan). QPCR was performed on the CFX96 Touch real-time PCR detection system (Bio-Rad, Hercules, CA, USA), using TB Green^®^ Premix Ex Taq II (Catalog No. RR820A, Takara). Relative mRNA expression was calculated using the 2Ct method with GAPDH as internal reference. Primer sequences for each target gene are listed in **Supplementary Table 2**.

### 2.14 Enzyme-linked immunosorbent assay (ELISA)

Cardiac tissue samples were homogenized in PBS to measure the inflammatory cytokine level. After centrifuge at 5,000*g* for 10 min at 4 °C, supernatants were collected. IL-1β, IL-18, and TNF-α were measured using specific ELISA kits (Mouse IL-1β ELISA Kit Catalog No. E-EL-M0037; Mouse IL-18 ELISA Kit Catalog No. E-EL-M0730; Mouse TNF-α ELISA Kit Catalog No. E-EL-M0049; Elabscience, Wuhan, China) according to manufacturer instructions. Absorption was measured at 450 nm using a microplate reader (Thermo Fisher Scientific).

### 2.15 Immunohistochemical staining (IHC)

Cardiac tissues were fixed in 4% paraformaldehyde (Catalog No. P0099, Beyotime, Shanghai, China), embedded in paraffin, and sectioned at 5 μm. For immunohistochemistry, sections underwent heat-induced antigen retrieval in citrate buffer (pH 6.0), and endogenous peroxidase activity was blocked with 3% H_2_O_2_. Sections were incubated with the appropriate primary antibodies overnight at 4 °C. Using the SP Link detection kit (Catalog No. SP-9000, ZSGB-BIO, Beijing, China), sections were then incubated sequentially with a biotinylated secondary antibody and streptavidin–horseradish peroxidase. Signals were visualized with the DAB peroxidase substrate kit (Catalog No. ZLI-9018, ZSGB-BIO, Beijing, China).

### 2.16 Statistical analysis

All data processing and statistical analysis were performed with R version 4.4.1 and GraphPad Prism 9.0. Data are represented as mean ± standard deviation. The Wilcoxon test or Student’s t-test was used to compare continuous variables between two groups. One-way or two-way analysis of variance followed by Bonferroni multiple comparisons were used to compare more than two groups. Pearson correlation coefficients were used to assess associations between variables. A *p*-value < 0.05 was considered statistically significant.

## 3 Results

**Figure 1:**
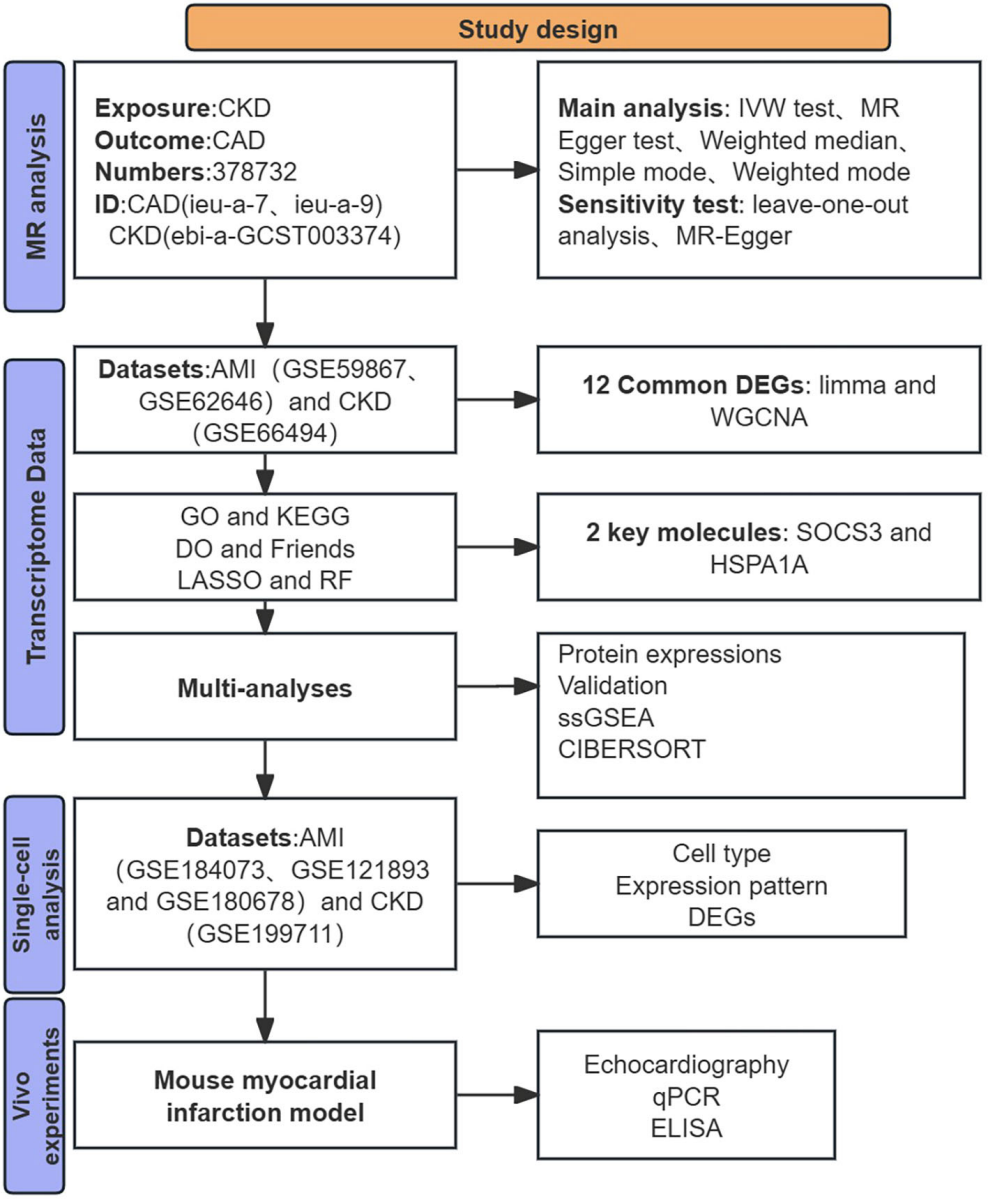
Flow chart of this study. First, MR showed that CKD is a risk factor for CAD. Second, transcriptomics data were used to search for genes shared by both diseases and associated with PANoptosis. Functional enrichment, immune microenvironment analysis, single-cell localization, and diagnostic performance validation of the candidate genes in a separate cohort of independent samples were carried out. Finally, gene loss experiments were carried out on animal models. Dataset selection based on (1) tissue relevance (PBMC/whole blood), (2) sample size >20 per group, (3) platform compatibility (Affymetrix U133 Plus 2.0), and (4) prior validation in peer-reviewed studies. Detailed sample sizes are provided in **Supplementary Table 1**.

The research design is summarized in **Figure 1**.

### 3.1 MR analysis of the causal relationship between CKD and CAD

We investigated potential causality between CKD and CAD using public GWAS data. First, SNPs robustly associated with CKD (*P* < 5 × 10^−8^) were selected from openGWAS as instrumental variables (IVs), and variants in linkage disequilibrium were pruned (*r*² < 0.001, distance < 10,000 kb). After filtering, the IV set for CAD outcomes comprised five SNPs (**Table 1**). The IVW analysis indicated that genetic liability to CKD had a significant positive causal effect on CAD risk (ieu-a-7: OR = 1.092, 95% CI: 1.014–1.176, *P* = 0.019; ieu-a-9: OR = 1.182, 95% CI: 1.044–1.339, *P* = 0.009; **Table 2**). This association is shown in the scatter plot (**Supplementary Figure 1A**). The weighted median method produced consistent results, supporting CKD as a potential risk factor for CAD.

**Table 1:** Characteristic of the CKD-related genetic variants and their effects on CAD (5 SNPs).

SNP	Chr	Position	EA	SNPs-CKD			SNPs-CAD		
				*β*	SE	*P*-value	*β*	SE	*P*-value
rs13333226	16	20365654	G	-0.21	0.02	3.80E-26	-0.020748	0.0114972	0.0711345
rs2453533	15	45641225	A	0.11	0.015	5.40E-12	-0.002498	0.0096146	0.795009
rs3812035	5	176817143	T	0.1	0.017	2.90E-09	0.015792	0.0105304	0.133702
rs7805747	7	151407801	A	0.14	0.019	2.10E-14	0.016746	0.011432	0.142965
rs13333226	16	20365654	G	-0.21	0.02	3.80E-26	-0.03511	0.013348	0.00854909

We conducted several sensitivity tests to test whether causal inference is robust. The heterogeneity test (Cochran’s *Q* = 2.234, *P* = 0.525) showed no significant heterogeneity among SNPs. The intercept test from MR-Egger regression yielded a *P*-value of 0.772 (> 0.05), indicating no clear horizontal pleiotropy in the instrumental variables (**Table 2**). Leave-one-out sensitivity analysis showed that removal of any single SNP did not significantly alter the overall estimate effect (**Supplementary Figures 1B, C**), which indicates the causal relationship was not driven by a single variant. Conclusion MR analyses support a strong genetic causal effect of CKD on CAD.

**Table 2:** Effect estimates of the associations between CKD and CAD.

Exposure GWAS ID	Outcome GWAS ID	Method	SNPs(N)	OR	95CI%	MR *P*-value	Heterogeneity Q/P-value	Pleiotropy *P*-value
CKD(ebi-a-GCST003374)	CAD(ieu-a-7)	IVW	4	1.092	1.014-1.176	0.019	2.234/0.525	
		MR Egger	4	1.139	0.879-1.476	0.429	2.117/0.347	0.772^a^
		Weighted median	4	1.109	1.016-1.21	0.021		
		Simple mode	4	1.126	0.997-1.276	0.153		
		Weighted mode	4	1.113	1.005-1.233	0.131		
	CAD(ieu-a-9)	Wald ratio	1	1.182	1.044-1.339	0.009		

CKD, chronic kidney disease; CAD, coronary artery disease; SNP, single nucleotide polymorphism; OR, odds ratio; CI, confidence interval; IVW, inverse-variance-weighted; MR, Mendelian randomization; ^a^, P-value of the intercept from MR Egger regression analysis.

### 3.2 Identification and functional analysis of shared gene signatures between AMI and CKD

To study possible associations between AMI and CKD, we included three RNA-seq datasets (GSE59867 and GSE62646 for AMI and GSE66494 for CKD) for systematic analysis. First, differential expression analysis (AMI screening criteria: |logFC| > 0.5 and adjusted *p*-value < 0.05; CKD screening criteria: |logFC| > 1 and adjusted *p*-value < 0.05) identified 164 DEGs in GSE59867 (**Supplementary Figures 2A**, **3A**), 402 DEGs in GSE62646 (**Supplementary Figures 2C**, **3B**), and 858 CKD-related DEGs in GSE66494 (**Supplementary Figures 2E**, **3C**). WGCNA identified key co-expression modules associated with disease phenotypes; the MEcyan module in GSE59867 (*r* = 0.62, **Supplementary Figures 2B**, **4A, B**) and the MEturquoise module in GSE62646 (*r* = 0.65; **Supplementary Figures 2D**, **4C, D**) were positively correlated with AMI, whereas the MEpink module in GSE66494 (*r* = 0.89; **Supplementary Figures 2F**, **4E, F**) was positively correlated with CKD.

**Figure 2:**
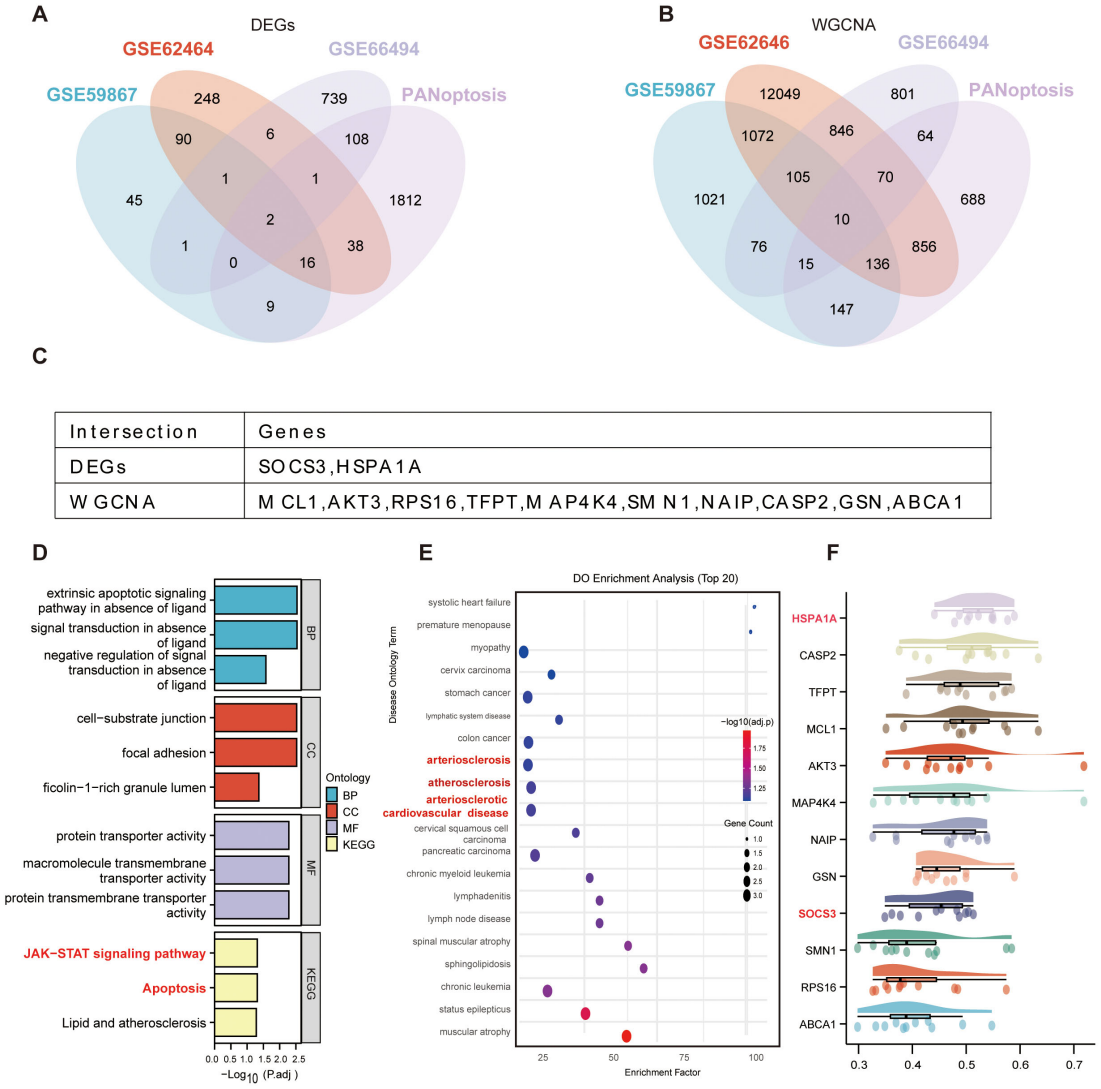
Shared gene signatures and functional enrichment among AMI, CKD, and PANoptosis. **(A)** Venn diagram showing two key DEGs shared by AMI (GSE59867, GSE62646), CKD (GSE66494), and PANoptosis. **(B)** Venn diagram showing 10 key module genes from positively correlated WGCNA modules in AMI (GSE59867, GSE62646), CKD (GSE66494), and PANoptosis. **(C)** List of genes shared between WGCNA modules and DEGs. **(D)** Bar chart of the top 12 significantly enriched GO and KEGG signaling pathways. **(E)** Bubble chart of the top 20 diseases from DO enrichment analysis. **(F)** Raincloud plot presenting Friends analysis of the core genes.

We intersected DEGs between AMI and CKD (**Figure 2A**) and key module genes identified by WGCNA (**Figure 2B**) with PANoptosis genes and found 12 common core genes (**Figure 2C**). Functional enrichment analysis showed that these 12 genes were primarily involved in ligand-independent signal transduction, apoptosis, the JAK–STAT signaling pathway, and lipid and atherosclerosis pathways (**Figure 2D**). Disease Ontology (DO) enrichment analysis indicated significant enrichment in cardiovascular diseases such as atherosclerosis (**Figure 2E**), suggesting their potential as biomarkers or therapeutic targets. In the protein–protein interaction network, HSPA1A and SOCS3 emerged as core genes (**Figure 2F**).

### 3.3 Screening and validation of shared diagnostic genes for AMI and CKD using machine learning algorithms

**Figure 3:**
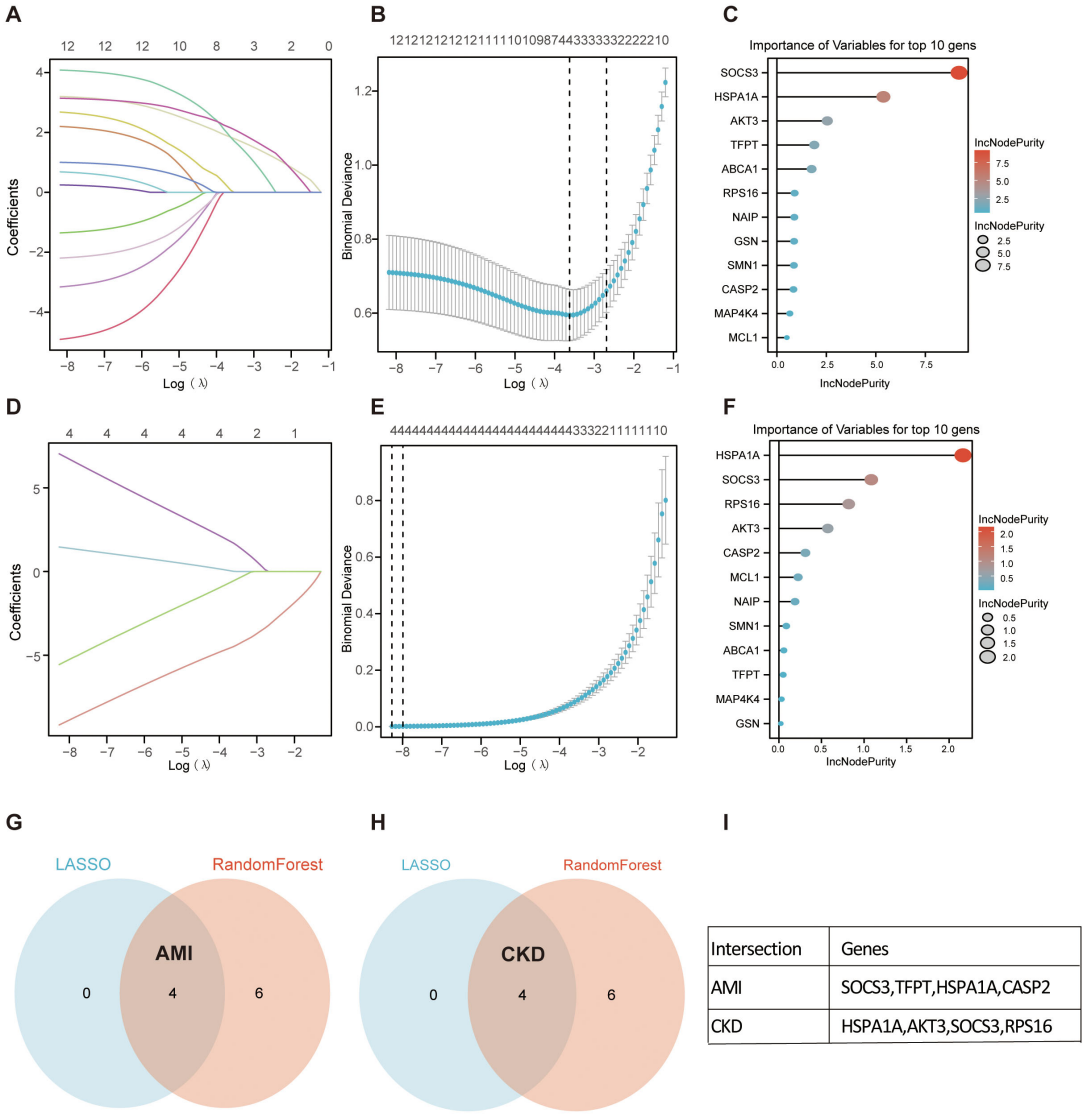
Identification of candidate biomarkers by machine learning. **(A, D)** Coefficient trajectories for variables in the LASSO regression model for AMI **(A)** and CKD **(D)**. **(B, E)** Tenfold cross-validation of tuning parameters for AMI **(B)** and CKD **(E)**. **(C, F)** Rankings of gene importance scores from the random forest algorithm for AMI **(C)** and CKD **(F)**. **(G, H)** Venn diagrams for AMI **(G)** and CKD **(H)** showing four hub genes obtained by intersecting results from LASSO regression and the random forest algorithm. **(I)** List of hub genes in AMI and CKD.

To refine the 12 core genes associated with AMI, CKD, and PANoptosis, we applied LASSO regression and an RF algorithm to identify final hub genes. LASSO selected four genes from the AMI-derived model, while the RF identified 10 genes (**Figures 3A–C**). Cross-analysis of these results yielded four candidate diagnostic genes for AMI (**Figures 3G, I**). In parallel, LASSO and RF, respectively, identified 4 and 10 CKD-related genes (**Figures 3D–F**). Cross-analysis of the CKD results produced four common candidate hub genes (**Figures 3H, I**).

After cross-comparison, SOCS3 and HSPA1A were identified as shared hub genes for AMI complicated by CKD (**Supplementary Figure 5A**). Validation analyses showed that in the AMI datasets (GSE59867 and GSE62646) and the validation set (GSE123342), SOCS3 and HSPA1A expressions were significantly upregulated in the disease groups (*P* < 0.001; **Supplementary Figures 5B, D, F, H**), and their AUCs for diagnosing AMI reached 0.916 and 0.866, respectively (**Supplementary Figures 6A, C**). In the CKD dataset (GSE66494), SOCS3 expression was upregulated while HSPA1A expression was downregulated (*P* < 0.001; **Supplementary Figures 5C, G**), yet both genes achieved AUCs of 1 for diagnosing CKD (**Supplementary Figures 6B, D**). Collectively, these results indicate that SOCS3 and HSPA1A have high diagnostic value for both AMI and CKD and are potential key biomarkers.

### 3.4 Biological functions and immune regulatory mechanisms of SOCS3 and HSPA1A

To elucidate the potential mechanisms by which the hub genes SOCS3 and HSPA1A act in AMI and CKD, we performed single-gene GSEA enrichment analysis and assessed immune microenvironment infiltration. GSEA showed that in AMI, SOCS3 was significantly enriched in upregulated pathways related to phagocytosis and lysosomal lumen (PHAGOCYTOSIS; LYSOSOMAL LUMEN) (**Supplementary Figures 6E**, **7A**). In CKD, SOCS3 was mainly enriched in upregulated immune and inflammatory pathways, including cytokine activity, neutrophil migration, and T-cell activation (**Supplementary Figures 6F**, **7B**). HSPA1A was also enriched in lysosome- and phagosome-related pathways in AMI, suggesting it functions through a “lysosome–phagocytosis–inflammation” regulatory axis (**Supplementary Figures 6G**, **7C**). In CKD, HSPA1A-enriched pathways related to membrane repolarization and renal system processes, indicating it may influence disease by regulating ion homeostasis and metabolic networks (**Supplementary Figures 6H**, **7D**).

**Figure 4:**
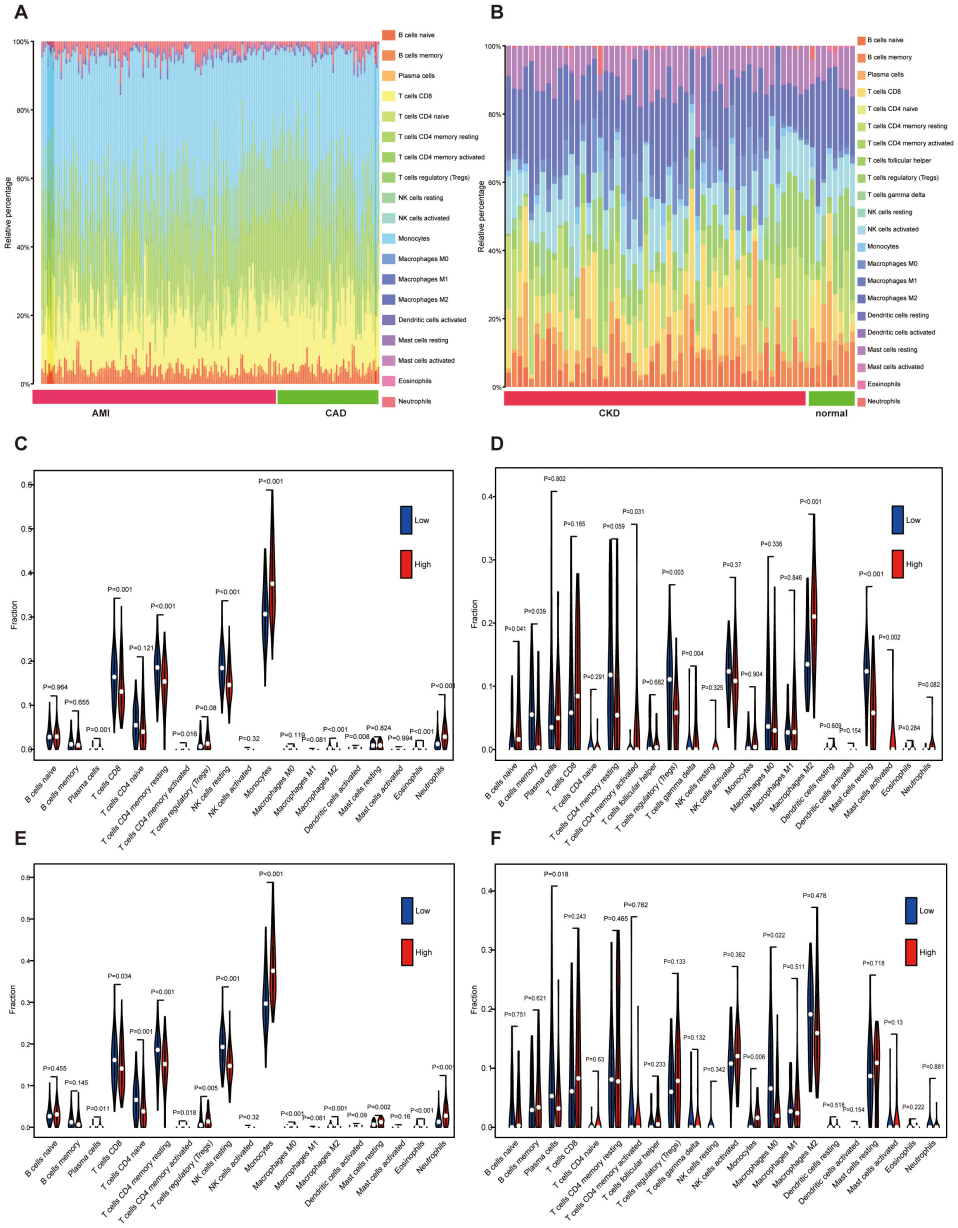
Immune cell infiltration and correlations with key immune-related genes. **(A, B)** Stacked bar charts of immune cell proportions in tissues from patients with AMI **(A)** and CKD **(B)**. **(C, D)** Box plots showing correlations between SOCS3 and various immune cell subsets in AMI **(C)** and CKD **(D)**. **(E, F)** Box plots showing correlations between HSPA1A and various immune cell subsets in AMI **(E)** and CKD **(F)**.

Functional enrichment analysis showed that patients with AMI and CKD were primarily enriched in immune response–related pathways. Therefore, we used CIBERSORT to evaluate immune cell infiltration in the datasets for both diseases. As shown in **Figures 4A, B**, monocytes comprised the largest proportion of cells in AMI samples, whereas macrophages comprised the largest proportion in CKD samples. Moreover, the proportions of these cell types were significantly higher in the disease groups than in the control groups (**Supplementary Figures 8A, B**). Correlation analysis indicated that, in AMI, SOCS3 and HSPA1A expression correlated positively and significantly with multiple immune cell subsets, including monocytes, neutrophils, and activated NK cells (**Figures 4C, E**; **Supplementary Figures 8C, E**). In CKD, these two genes correlated positively and significantly with macrophages, T cells, mast cells, and others (**Figures 4D, F**; **Supplementary Figures 8D, F**). Collectively, these results suggest that SOCS3 and HSPA1A may play key roles in the pathogenesis of AMI and CKD by regulating the infiltration and function of specific immune cells.

### 3.5 Construction of cell atlases for CAD and CKD using scRNA-seq data

**Figure 5:**
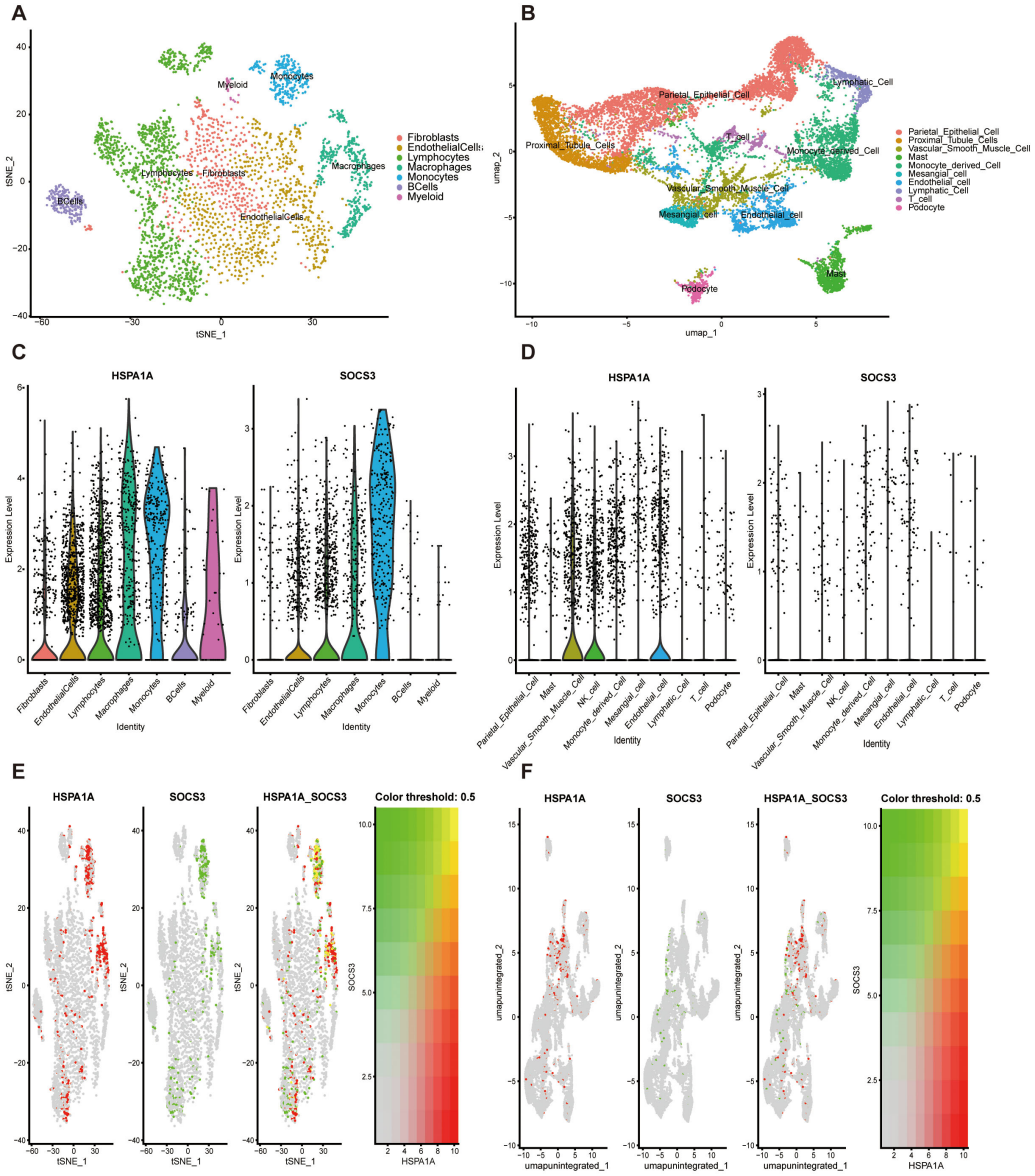
Single-cell transcriptional profiles in patients with AMI and CKD. **(A)** UMAP representation of 4,400 cells from the AMI scRNA-seq dataset. **(B)** UMAP representation of 12,837 cells from the CKD scRNA-seq dataset. **(C, D)** Violin plots of SOCS3 and HSPA1A expression across cell populations in AMI **(C)** and CKD **(D)**. **(E, F)** Single-cell co-localization of SOCS3 and HSPA1A in AMI **(E)** and CKD **(F)**.

Because the preceding analyses used bulk transcriptomics and did not capture the cellular heterogeneity of the vascular microenvironment in AMI and CKD at single-cell resolution, we integrated single-cell RNA sequencing (scRNA-seq) data from patients with CAD (GSE184073, GSE180678) and CKD (GSE121893, GSE199711) for deeper analysis. CAD samples were obtained from three stable CAD patients and two AMI patients. After stringent quality control and normalization, 4,400 high-quality cells remained in the CAD dataset and 12,837 high-quality cells remained in the CKD dataset. We identified highly variable genes and performed unsupervised clustering to construct disease-specific cell atlases. Using established marker genes, we annotated seven major cell types in the CAD data, including fibroblasts, endothelial cells, lymphocytes, macrophages, and monocytes (**Figure 5A**), and annotated ten cell types in the CKD data, including parietal epithelial cells, proximal tubular cells, vascular smooth muscle cells, and mast cells (**Figure 5B**). The marker gene expression patterns validated the accuracy of these annotations (**Supplementary Figures 9A, B**).

### 3.6 Single-cell expression characteristics of core genes SOCS3 and HSPA1A

**Figure 6:**
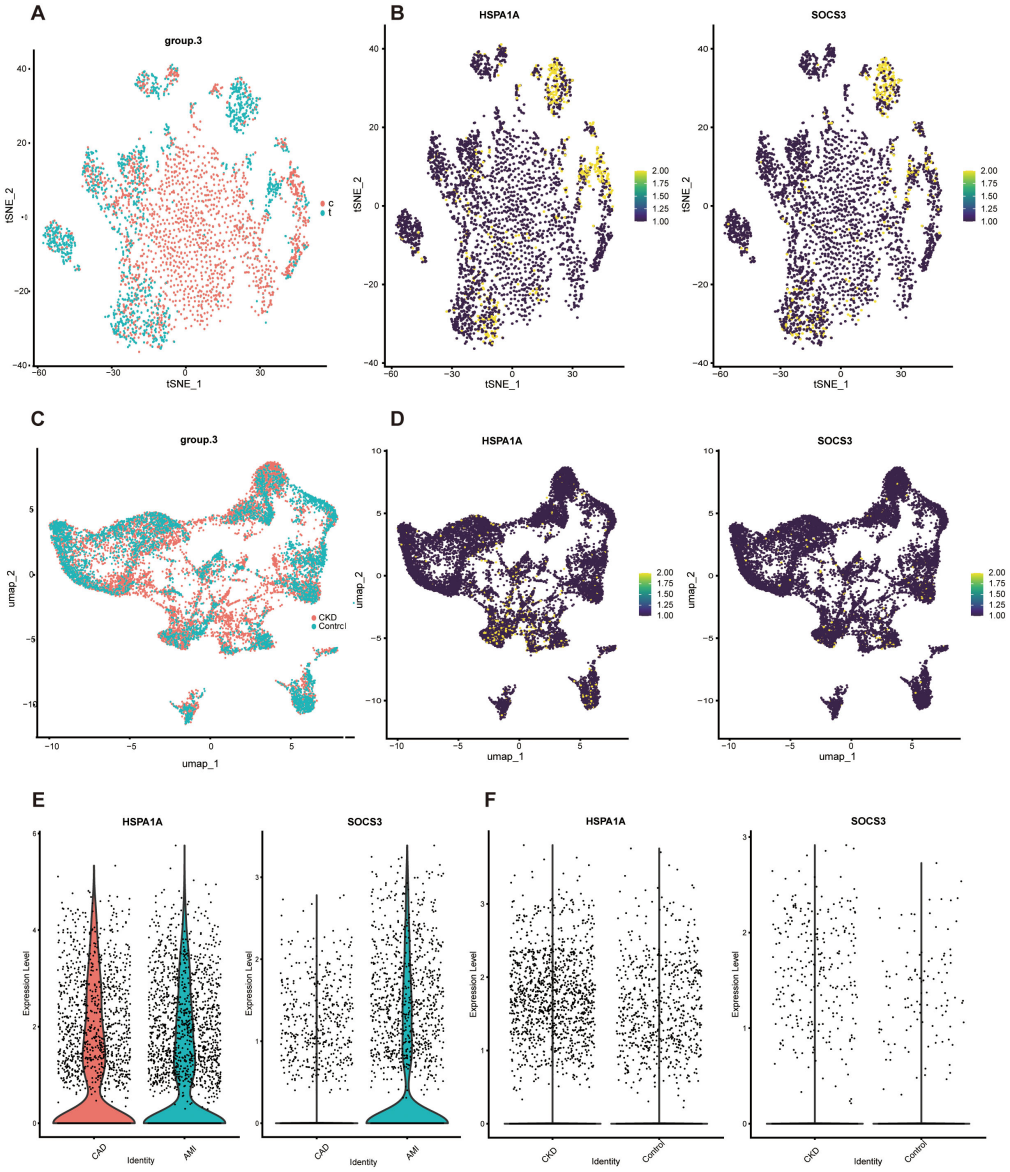
Single-cell transcriptional features of disease and control groups in AMI and CKD. **(A, C)** UMAP plots showing cell group distributions in AMI **(A)** and CKD **(C)**. **(B, D)** UMAP overlays of SOCS3 and HSPA1A expression in AMI **(B)** and CKD **(D)**. **(E, F)** Violin plots of SOCS3 and HSPA1A expression in AMI **(E)** and CKD **(F)**.

**Figure 7:**
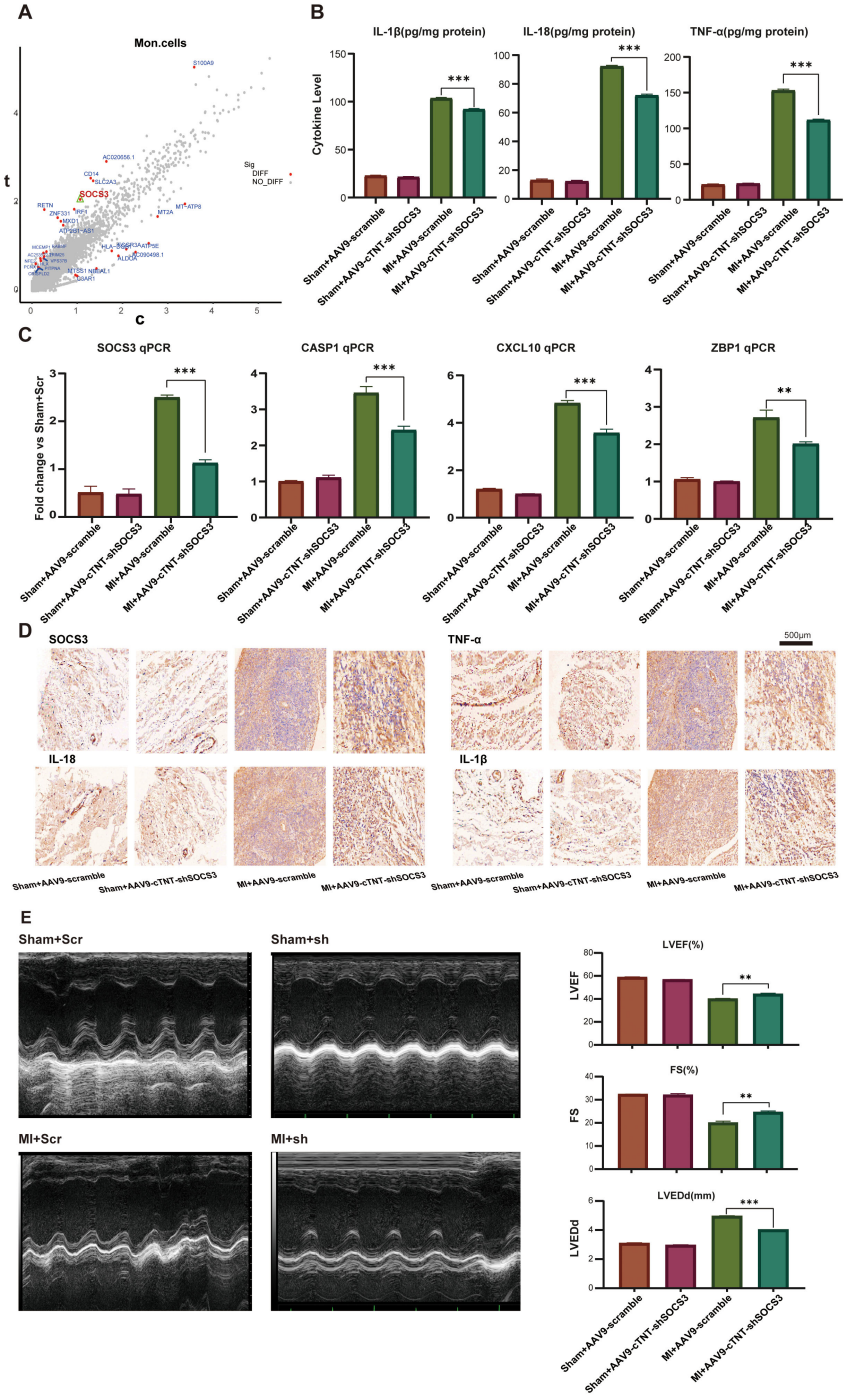
SOCS3 knockdown reduces MI-induced PANoptosis and improves cardiac function. **(A)** SOCS3 expression differed across monocyte subsets in AMI. **(B)** ELISA showed that secretion of key inflammatory cytokines (IL-18, IL-1β, and TNF-α) increased significantly in the MI group. **(C)** qPCR revealed that MI surgery significantly elevated mRNA levels of Casp1, Cxcl10, Zbp1, and SOCS3 in the MI + AAV9-scramble group. **(D)** Immunohistochemical staining assessed the expression of SOCS3, IL-18, IL-1β, and TNF-α proteins in mouse heart tissues (scale bar: 500 μm). **(E)** Echocardiography indicated that ejection fraction and fractional shortening were higher in SOCS3-knockdown mice than in the MI + AAV9-scramble group. ** : P < 0.01, *** : P < 0.001

Using the cell atlas described above, we evaluated the single-cell expression patterns of two core genes, SOCS3 and HSPA1A. In the complex microenvironment of CAD and CKD, SOCS3 and HSPA1A were highly expressed mainly in endothelial cells and monocytes/monocyte-derived cells (**Figures 5C, D**). Notably, these genes were co-expressed within the same monocyte population (**Figures 5E, F**), suggesting potential functional synergy. Consistent with the bulk transcriptome analysis, single-cell data also showed significant enrichment of SOCS3 and HSPA1A expression in specific cell types from disease-group samples (**Figures 6A–F**), further supporting their likely roles in disease pathogenesis. Detailed analysis revealed that SOCS3 was a key DEG in monocytes from AMI patients (**Figure 7A**), providing cellular evidence for its role as a core driver of AMI pathogenesis.

### 3.7 Knockdown of SOCS3 reduced PANoptosis induced by myocardial infarction and improved cardiac function

To investigate the function of SOCS3 in MI, we specifically knocked down its expression in cardiomyocytes using adeno-associated virus type 9 (AAV9) encoding a short hairpin RNA targeting SOCS3 (AAV9-cTNT-shSOCS3). Mice were assigned to four groups: sham + AAV9-scramble, sham + AAV9-cTNT-shSOCS3, MI + AAV9-scramble, and MI + AAV9-cTNT-shSOCS3.

The knockdown efficiency of SOCS3 was verified by qPCR (**Figure 7C**). Compared with the control group, SOCS3 expression was significantly reduced in the MI + AAV9-cTNT-shSOCS3 group. Because PANoptosis plays a novel role in cardiac injury, we further examined key PANoptosis-related markers. qPCR analysis (**Figure 7C**) showed that MI significantly upregulated mRNA levels of CASP1, CXCL10, and ZBP1 (indicating activation of the p-RIPK3 pathway), as well as SOCS3 itself, in the MI + AAV9-scramble group. Notably, SOCS3 silencing effectively attenuated the MI-induced upregulation of these proinflammatory and PANoptosis-associated genes. Consistent with these findings, ELISA results (**Figure 7B**) demonstrated that secretion of key inflammatory cytokines (IL-18, IL-1β, and TNF-α) was markedly increased in the MI + AAV9-scramble group but reduced in the MI + AAV9-cTNT-shSOCS3 group. Immunohistochemical staining (**Figure 7D**) further confirmed that SOCS3 knockdown inhibited accumulation of SOCS3 and PANoptosis-related proteins in the infarcted myocardium.

We then evaluated whether SOCS3 knockdown protected cardiomyocyte survival and cardiac function. Echocardiography (**Figure 7E**) showed that, although MI caused severe cardiac dysfunction, mice with SOCS3 knockdown exhibited significantly improved myocardial contractility, as indicated by increased ejection fraction and fractional shortening compared with the MI + AAV9-scramble group.

## 4 Discussion

Our goal was to investigate how AMI risk for patients with CKD is largely explained by chronic inflammation. Most commonly, the risk of AMI and CKD is not fully explained by the traditional risk factors, and chronic inflammation is considered the link between the two diseases. We analyzed the DEGs and signaling pathways shared by AMI and CKD. The molecular signatures associated with PANoptosis were significantly enhanced, suggesting that this new form of inflammatory PCD may play a role in the interaction between the two diseases. PANoptosis combines the features of apoptosis, pyroptosis, and necroptosis and can be activated by specific molecular complexes such as PANoptosomes.

We hypothesize that in CKD accumulated uremic toxins and long-term oxidative stress trigger PANoptosis by activating the NLRP3 inflammasome and related pathways. In the vasculature, the NLRP3 inflammasome accelerates endothelial injury and increases the release of proinflammatory mediators, leading to atheroma formation and reducing plaque stability. In the myocardium, PANoptosis can directly lead to the widespread inflammatory death of cardiomyocytes and worsen ischemia–reperfusion injury. This provides a new molecular perspective of the cardiorenal interaction. PANoptosis may serve as a major effector by which CKD drives systemic inflammation, leading to atherosclerosis and AMI. Targeting critical nodes of the PANoptosis pathway could provide new therapeutics to break the cycle and slow the progression of CAD.

We identified a significant causal relationship between CKD and CAD using MR analysis, indicating that CKD is a risk factor for CAD. This result aligns with epidemiological studies showing that CKD independently increases cardiovascular risk even after adjustment for traditional risk factors such as hypertension and dyslipidemia. Sensitivity analyses such as Cochran’s Q test and MR-Egger regression confirmed the robustness of our results except, for heterogeneity and horizontal pleiotropy. These results highlight the importance of monitoring and managing CKD in order to reduce CAD and other complications such as AMI.

Transcriptomic analysis identifies DEGs and co-expression modules associated with AMI and CKD. Notably, the intersection of DEGs and co-expression modules with PANoptosis-related genes reveals 12 key genes, which are further validated as diagnostic biomarkers. These genes are linked to critical pathways, including the JAK-STAT signaling pathway, apoptosis, and lipid metabolism. The JAK-STAT pathway serves as a crucial signal transduction mechanism mediated by cytokines. Apoptosis represents the primary form of PCD. Additionally, the lipid and atherosclerosis pathway encompasses lipid metabolism disorders, oxidative stress, and vascular inflammation. All three conditions (AMI, CKD, and PANoptosis) are associated with inflammation (through the JAK-STAT pathway), apoptosis, and metabolic disorder. The activation of the JAK-STAT pathway facilitates the release of inflammatory factors, which in turn accelerates tissue damage and fibrosis. The activation of the apoptosis pathway causes the loss of myocardial cells and renal cells and further damages organ function. The abnormal metabolic process of the lipids causes AMI and leads to apoptosis and inflammation through oxidative stress. These pathways are central to the inflammation and metabolic dysregulation observed in AMI and CKD. This finding provides a theoretical basis for understanding the comorbidity mechanisms of these diseases and developing broad-spectrum treatment strategies. This aligns with previous reports indicating that systemic inflammation is a key driver of cardiovascular disease, particularly in patients with CKD (Zewinger et al., 2016; Hansson, 2017).

Our study revealed the crucial role of PANoptosis in AMI and CKD, highlighting its significance as a shared core mechanism for these two conditions. PANoptosis is not a single mode of cell death but integrates characteristic features of apoptosis, pyroptosis, and necrosis. This complex interplay likely accounts for the extensive tissue damage observed in AMI and CKD. Through systematic bioinformatics analysis, we identified key genes closely associated with the PANoptosis process, providing a novel framework for understanding how multiple cell-death pathways are synergistically activated and drive disease progression to severe stages. Based on this mechanism, developing targeted interventions against PANoptosis execution components (e.g., specific caspase family members or GSDM proteins) was expected to be a promising therapeutic approach and opened new avenues for drug development to mitigate cardiac and renal injury caused by AMI and CKD.

We further identified two upregulated targets, SOCS3 and HSPA1A, using machine learning methods. Their upregulation in the disease group indicates potential roles in regulating immune responses and cellular stress, which further exacerbated tissue damage. Suppressors of cytokine signaling (SOCS) are a family of negative regulatory proteins that precisely control cytokine signal transduction by inhibiting the Janus kinase/signal transducer and activator of transcription (JAK/STAT) pathway (Yoshimura et al., 2021). The family comprises eight highly conserved members—SOCS1–7 and the cytokine-inducible SH2-containing protein (CIS)—which share similar structures and functions. These proteins primarily compete for phosphorylated tyrosine sites on JAK kinases or cytokine receptors, thereby blocking STAT phosphorylation and subsequent nuclear translocation (Carow and Rottenberg, 2014). SOCS3, a prominent family member, plays a central role in regulating inflammation; it potently inhibits interleukin-6 (IL-6) and granulocyte colony-stimulating factor (G-CSF)–mediated inflammatory responses and thus serves as a key regulator in various inflammatory diseases (Hu et al., 2021).

As a principal negative regulator of JAK/STAT signaling, SOCS3—and its mimetics or antagonists—has strong potential as a novel intracellular checkpoint modulator (Yoshimura and Yasukawa, 2012; La Manna et al., 2020). KEGG pathway enrichment analysis showed that the SOCS3 gene was significantly enriched in the JAK–STAT signaling pathway, suggesting that SOCS3 may influence the disease processes of AMI and CKD by modulating this pathway. scRNA-seq further revealed that SOCS3 was highly expressed in monocytes and also displayed notable expression in endothelial cells, with a pronounced differential expression pattern in monocytes from AMI patients. Together, these results indicate that SOCS3 may be a pathogenic biomarker and therapeutic target for AMI and CKD—especially AMI—offering new directions for diagnosis and treatment.

Heat shock protein family A member 1A (HSPA1A), encoded by the HSPA1A gene and also called inducible HSP70 or HSP72, is a major cytoprotective HSP70 protein (Wu et al., 2021). HSPA1A is a 70-kDa molecular chaperone that is essential for the cellular stress response (Lindquist and Craig, 1988; Bukau and Horwich, 1998). It primarily functions in the cytosol but also translocates to the plasma membrane of heat-shocked and cancer cells. HSPA1A expression increases in many pathological conditions, and induction of HSPA1A has effectively slowed the progression of several diseases (Hu et al., 2022). Our transcriptomic analysis confirmed that HSPA1A is highly expressed in AMI and CKD and is closely associated with both conditions. Further investigation is needed to determine whether CKD promotes AMI progression by activating HSPA1A.

The suppressor of cytokine signaling SOCS3 functions as a negative feedback regulator of excessive inflammation, while the heat shock protein HSPA1A protects cells during stress. GSEA showed that these genes are enriched in pathways related to phagocytosis, lysosomal function, and immune cell activation, highlighting their roles in disease pathogenesis. SOCS3, a canonical inhibitor of cytokine signaling (e.g., the JAK–STAT pathway), was upregulated in pathways such as PHAGOCYTOSIS and LYSOSOMAL LUMEN, which likely reflects negative feedback control of excessive inflammatory signals (e.g., TNF-α and IL-6) and coordination of phagocytic clearance with protein synthesis. In CKD, SOCS3 upregulation in pathways associated with immune–inflammatory regulation (e.g., cytokine activity, neutrophil migration, and T-cell activation), leukocyte proliferation and adhesion, and disrupted acid–base balance suggests that SOCS3 contributed to inflammation–immune imbalance and complication development during CKD progression by limiting excessive immune activation, modulating leukocyte function, and helping to maintain metabolic homeostasis. The overlap of these pathways in AMI and CKD indicates a shared mechanistic basis for their comorbidity, driven by chronic inflammation and dysregulated cell death.

Immunoinfiltration analysis showed significant differences in monocyte and macrophage abundance between the disease and control groups, with strongly related expressions of SOCS3 and HSPA1A. Single-cell RNA sequencing mapped these genes to monocytes and endothelial cells, suggesting they may participate in immune cell recruitment and vascular dysfunction. Coexpression of SOCS3 and HSPA1A in monocytes indicates a synergistic role in regulating the inflammatory response, which may be crucial to the progression of AMI and CKD.

Our study identified two PANoptosis genes, SOCS3 and HSPA1A, as comorbid genes associated with AMI and CKD, providing new insights into the pathogenesis of both diseases. SOCS3 and HSPA1A could be targets for developing precision medicine approaches for early detection and targeted treatment of AMI and CKD patients. In single-cell data analysis, SOCS3 was identified as a DEG in monocytes from the AMI group, suggesting that SOCS3 merits further investigation as a core gene in AMI pathogenesis. Recent studies have shown that SOCS3 is not only a standard negative feedback regulator of JAK/STAT but also a “cell death signal integrator” in innate immune cells, particularly macrophages and monocytes. It determines cell fate (pro-inflammatory death or survival) directly by interaction with PANoptosome components (such as NLRP3, ASC, and ZBP1) and by controlling assembly and activity of these components (Zhang et al., 2020; Morelli et al., 2024).

The results of the animal experiments in this study more directly demonstrated the pathological role of SOCS3. In a mouse model, cardiac-specific knockdown of SOCS3 significantly reduced the expression of key PANoptosis effector proteins, including activated CASP1, ZBP1, and CXCL10, and markedly improved cardiac function. This study was the first to clarify at the molecular level the positive regulatory role of SOCS3 in PANoptosis during ischemic cardiac injury, identifying a novel molecular target and opening a new research direction for understanding the complex interactions between cell death and immune responses in CAD.

Although our study indicates that SOCS3 and HSPA1A have good diagnostic performance and clinical value in patients with AMI and CKD, the study has several limitations. This study has several limitations. First, the GWAS summary data used for the MR analysis did not include a cohort specifically composed of patients with comorbid CKD and CAD. Although we searched for independent datasets that contained both CKD and CAD phenotypes, such data were scarce. Nonetheless, large-scale studies have established a causal relationship between CKD and CAD. Sarnak et al. identified CKD as an independent risk factor for cardiovascular disease (Sarnak et al., 2003), and Go et al. showed that CKD is associated with an increased risk of cardiovascular events and death (Go et al., 2004). Second, the dataset sample size is limited, especially for CKD patients, and the high AUC value may result from insufficient data. Reliance on public datasets may also introduce biases related to sample heterogeneity and demographics. Third, the cross-sectional nature of some analyses limited our ability to infer temporal relationships between gene expression changes and disease progression. Recent studies show that PANoptosis regulation is complex, and other factors such as IRF1 and AIM2 also play important roles (Nadella and Kanneganti, 2024). We did not know whether SOCS3 controls PANoptosome via protein–protein interactions, ubiquitination, or transcriptional regulation. In addition, the animal model only used cardiac-specific knockdown and did not reproduce the comorbid condition of AMI on a CKD background. Systemic features of CKD (uremic toxins, calcium–phosphorus imbalances) may affect SOCS3 basal expression and function. Although machine learning has identified HSPA1A as a shared core gene for CKD and AMI, its regulatory role in PANoptosis remains unclear. Future studies are needed to investigate the mechanism by which HSPA1A mediates PANoptosis-related cardiac injury, including its potential interaction with the SOCS3 signaling pathway.

Future studies should validate results in comorbid models, assess SOCS3-targeted treatments in CKD-AMI composite models, describe specific biochemical pathways by which SOCS3 regulates PANoptosis, and develop specific cell type–selective SOCS3 treatments to maximize efficacy and minimize systemic side effects.

Single-cell transcriptome analysis of the AAV-mediated SOCS3 knockdown mouse model would provide in-depth insights into cell type–specific transcriptomic changes and immune cell heterogeneity, reveal the impact of SOCS3 deficiency on cardiac cell populations, and identify potential therapeutic targets. Due to current limitations in technology and resources, we did not perform this analysis. As single-cell technologies become more widespread, this approach will become an important future research direction.

## 5 Conclusion

In summary, this study used MR, bulk expression profiling, scRNA-seq, and animal experiments to characterize patients with AMI and CKD. The comprehensive analysis elucidated the shared genetic and mechanistic basis of AMI and CKD, identifying PANoptosis as a key link between the two diseases. SOCS3, significantly upregulated in AMI patient monocytes and functionally validated in our AMI mouse model, represents a primary therapeutic target. In contrast, HSPA1A, identified as a hub gene through machine learning, remains a secondary candidate requiring further functional validation. These findings deepened our understanding of the complex interplay between cardiovascular and kidney disease and paved the way for future research aimed at improving clinical outcomes for affected patients.

## Data Availability

Publicly available datasets were analyzed in this study. This data can be found here: https://www.ncbi.nlm.nih.gov/geo/. The accession numbers are GSE59867, GSE62646, GSE66494, GSE123342, GSE184073, GSE121893, GSE180678 and GSE199711.
