# Supplementary material for: Exploration of SOCS3 and HSPA1A in PANoptosis during acute myocardial infarction and chronic kidney disease
Source: Front Cell Infect Microbiol. 2026 Jul 22;16:1844856. doi: 10.3389/fcimb.2026.1844856 (PMC13437532; doi:10.3389/fcimb.2026.1844856)
Supplement: Supplementary file 1 [file Table1.docx]

**Figure legends**

**Figure S1.** **The causality of CKD on CAD risk.** (A) Scatter plot. The slope of each line represents the estimated effect from the corresponding MR method. Symmetry in the funnel plot indicates no obvious horizontal pleiotropy. IVW, inverse variance weighted; SNP, single-nucleotide polymorphism. (B) Forest plot. The red points show integrated estimates obtained by combining all SNPs using the IVW method. Horizontal lines indicate 95% confidence intervals. (C) Leave-one-out analysis. Black points show IVW estimates obtained after excluding each specific variant in turn. The red point denotes the IVW estimate using all SNPs.

**Figure S2. Differential expression analysis and co-expression modules in AMI and CKD samples.** (A, C, E) Volcano plots indicating DEGs in AMI (GSE59867, GSE62646) and CKD (GSE66494) samples. (B, D, F) Heatmaps showing the associations between modules and clinical traits in AMI (GSE59867, GSE62646) and CKD (GSE66494).

**Figure S3. Heat maps of DEGs in AMI and CKD samples.** (A–C) Heat maps showing DEGs in AMI (GSE59867, GSE62646) and CKD (GSE66494) samples; the top 20 genes are highlighted.

**Figure S4. WGCNA analysis of AMI and CKD. (**A, C, E) The selection of soft-thresholding powers for AMI (GSE59867, GSE62646) and CKD (GSE66494). (B, D, F) Gene clustering dendrograms for AMI and CKD constructed using the dynamic tree-cutting algorithm.

**Figure S5. Diagnostic value of core genes HSPA1A and SOCS3.** (A)Venn diagram showing two hub genes common to AMI and CKD. (B, C) Expression levels of SOCS3 in the AMI (B) and CKD (C) datasets. (D, E) Expression levels of SOCS3 and corresponding ROC curve analyses in the validation dataset. (F, G) Expression levels of HSPA1A in the AMI (F) and CKD (G) datasets. (H, I) Expression levels of HSPA1A and corresponding ROC curve analyses in the validation dataset.

**Figure S6. Validation of SOCS3 and HSPA1A and GSEA enrichment plots.** (A,C) AUC values for SOCS3 and HSPA1A in AMI. (B,D) AUC values for SOCS3 and HSPA1A in CKD. (E,F)GSEA enrichment plots for SOCS3 in AMI and CKD. (G,H) GSEA enrichment plots for HSPA1A in AMI and CKD.

**Figure S7. Faceted enrichment bubble plots from GSEA for SOCS3 and HSPA1A.** (A) Faceted GSEA bubble plot for SOCS3 in AMI. (B) Faceted GSEA bubble plot for SOCS3 in CKD. (C) Faceted GSEA bubble plot for HSPA1A in AMI. (D) Faceted GSEA bubble plot for HSPA1A in CKD.

**Figure S8. Immune cell infiltration in the two groups and its correlation with SOCS3 and HSPA1A.** (A, B) Box plots showing correlations among immune cell subsets in disease and control groups for AMI (A) and CKD (B). (C, E) Lollipop plots showing correlations between SOCS3 (C) and HSPA1A (E) and various immune cell subsets in AMI. (D, F) Lollipop plots showing correlations between SOCS3 (D) and HSPA1A (F) and various immune cell subsets in CKD.

**Figure S9. Classic marker genes for AMI and CKD.** A, Classic marker genes for AMI. B, Classic marker genes for CKD.
