# Supplementary material for: Exploration of SOCS3 and HSPA1A in PANoptosis during acute myocardial infarction and chronic kidney disease
Source: Front Cell Infect Microbiol. 2026 Jul 22;16:1844856. doi: 10.3389/fcimb.2026.1844856 (PMC13437532; doi:10.3389/fcimb.2026.1844856)
Supplement: Supplementary file 2 [file Table2.docx]

Supplementary Table S1 The details of the eight datasets which we had selected.

| Rank | Accession number | Platform | Disease group | control group | Organ |
| --- | --- | --- | --- | --- | --- |
| 1 | GSE59867 | GPL6244 | 111 | 46 | peripheral blood mononuclear cells |
| 2 | GSE62646 | GPL6244 | 28 | 14 | peripheral blood mononuclear cells |
| 3 | GSE66494 | GPL6480 | 53 | 8 | Renal biopsy specimens |
| 4 | GSE123342 | GPL17586 | 65 | 22 | whole blood |
| 5 | GSE184073 | GPL24676 | 1 | 1 | Coronary Atherosclerotic Plaques |
| 6 | GSE121893 | GPL18573 | 0 | 2 | heart |
| 7 | GSE180678 | GPL20301 | 1 | 0 | Myocardium (Left Ventricle) |
| 8 | GSE199711 | GPL24676 | 3 | 2 | Kidney |

Supplementary Table S2 Primer Sequences Used for qRT-PCR Analysis.

|  | forward primer | reverse primer |
| --- | --- | --- |
| SOCS3 | CACTCTTCAGCATCTCTGTCG | CTCCTGCACCTTTGTAGTCC |
| CASP1 | GGCACATTTCCAGGACTGACTG | GCAAGACGTGTACGAGTGGTTG |
| CXCL10 | ATCATCCCTGCGAGCCTATCCT | GACCTTTTTTGGCTAAACGCTTTC |
| ZBP1 | GATCTACCACTCACGTCAGGAAG | GGCAATGGAGATGTGGCTGTTG |

Supplementary Table S3 The list of abbreviations.

| Rank | Full name | List of abbreviations | Rank | Full name | List of abbreviations |
| --- | --- | --- | --- | --- | --- |
| 1 | Acute myocardial infarction | AMI | 19 | Random Forest | RF |
| 2 | Chronic kidney disease | CKD | 20 | Gene set enrichment analysis | GSEA |
| 3 | Coronary artery disease | CAD | 21 | Highly variable genes | HVGs |
| 4 | Glomerular filtration rate | GFR | 22 | Principal component analysis | PCA |
| 5 | Programmed cell death | PCD | 23 | specific pathogen free | SPF |
| 6 | Mendelian randomization | MR | 24 | Left ventricular ejection fraction | LVEF |
| 7 | Genome-wide association study | GWAS | 25 | Left ventricular fractional shortening | LVFS |
| 8 | Single nucleotide polymorphisms | SNPs | 26 | quantitative real time PCR | qPCR |
| 9 | Inverse variance weighting | IVW | 27 | Enzyme-linked immunosorbent assay | ELISA |
| 10 | Odds ratio | OR | 28 | Instrumental variables | IVs |
| 11 | Linear Models for Microarray Data | LIMMA | 29 | Linkage disequilibrium | LD |
| 12 | Differentially expressed genes | DEGs | 30 | Disease Ontology | DO |
| 13 | Weighted gene co-expression network analysis | WGCNA | 31 | Receiver operating characteristic | ROC |
| 14 | Gene significance | GS | 32 | Area under curve | AUC |
| 15 | Module membership | MM | 33 | Suppressors of cytokine signaling | SOCS |
| 16 | Gene ontology | GO | 34 | Interleukin-6 | IL-6 |
| 17 | Kyoto Encyclopedia of Genes and Genomes | KEGG | 35 | Granulocyte colony-stimulating factor | G-CSF |
| 18 | Least Absolute Shrinkage and Selection Operator | LASSO | 36 | Heat shock protein family A member 1A | HSPA1A |
